# Atypical Presentation of Insulinoma in a Patient With Hypoglycemia Unawareness: A Case Report

**DOI:** 10.7759/cureus.83789

**Published:** 2025-05-09

**Authors:** Arowa Abdelgadir, Jimmy Li Voon Chong

**Affiliations:** 1 Internal Medicine, Royal Hampshire County Hospital, Winchester, GBR; 2 Diabetes and Endocrinology, Royal Hampshire County Hospital, Winchester, GBR

**Keywords:** hypoglycemia, hypoglycemic unawareness, neuroendocrine tumour, pancreatic insulinoma, whipple's procedure

## Abstract

Insulinomas are uncommon tumors in the pancreas that result in the overproduction of insulin, which can cause frequent episodes of low blood sugar. While most patients present with typical symptoms such as confusion and diaphoresis, some may experience hypoglycemia unawareness. This occurs when repeated episodes of low blood glucose impair the body’s autonomic responses, making it difficult for the patient to recognize early signs of impending hypoglycemia. As a result, diagnosis can be delayed, increasing the risk of severe complications such as seizures, coma, or accidents. We present the case of a 69-year-old male diagnosed with insulinoma after a motor vehicle accident, which revealed a dangerously low blood glucose level of 2.8 mmol/L, despite the patient reporting no symptoms. Further investigations, including a supervised 72-hour fast, showed abnormally high C-peptide (1,350 pmol/L) and insulin (14 mU/L) levels, with a lab glucose reading of 2.2 mmol/L. A CT scan of the pancreas revealed a 1.25 cm enhancing lesion in the proximal body, consistent with an insulinoma. The patient underwent a successful Whipple procedure, and his postoperative recovery was uneventful. This case highlights the diagnostic challenges of insulinomas, especially in patients with hypoglycemia unawareness. It underscores the importance of considering insulinoma in the differential diagnosis of patients presenting with hypoglycemia unawareness. Early recognition and intervention are crucial to prevent serious complications associated with this condition.

## Introduction

Insulinomas are rare pancreatic neuroendocrine tumors characterized by excessive insulin production, leading to a range of hypoglycemic symptoms. Most patients present with features consistent with Whipple’s triad: symptomatic hypoglycemia, documented low blood glucose, and resolution of symptoms following glucose administration. However, a subset of patients may develop hypoglycemic unawareness due to adaptive blunting of autonomic responses resulting from chronic or recurrent hypoglycemia [1].

When hypoglycemic unawareness is the presenting feature in insulinoma patients, it poses a unique diagnostic challenge, as these individuals may not recognize early warning signs such as sweating, tremors, or palpitations. This can result in delayed diagnosis and an increased risk of serious complications, including loss of consciousness, seizures, or injury from accidents [2].

Here, we report the case of a 69-year-old male diagnosed with insulinoma following a car accident, during which he was found to be hypoglycemic.

## Case presentation

A 69-year-old male presented to the emergency department following a motor vehicle accident. On the day of the incident, he had consumed breakfast but skipped lunch. Pre-hospital blood glucose measurement by the ambulance crew revealed a level of 2.8 mmol/L, although the patient denied any symptoms suggestive of hypoglycemia and had no recollection of the events leading up to the accident. Notably, he has never experienced symptoms such as light-headedness, fainting, tremors, or sweating.

The patient’s medical history includes osteoarthritis of the knee and hypercholesterolemia. He was taking atorvastatin 20 mg daily and had no known drug allergies. Additionally, there is no family history of diabetes or endocrine disorders.

**Figure 1:**
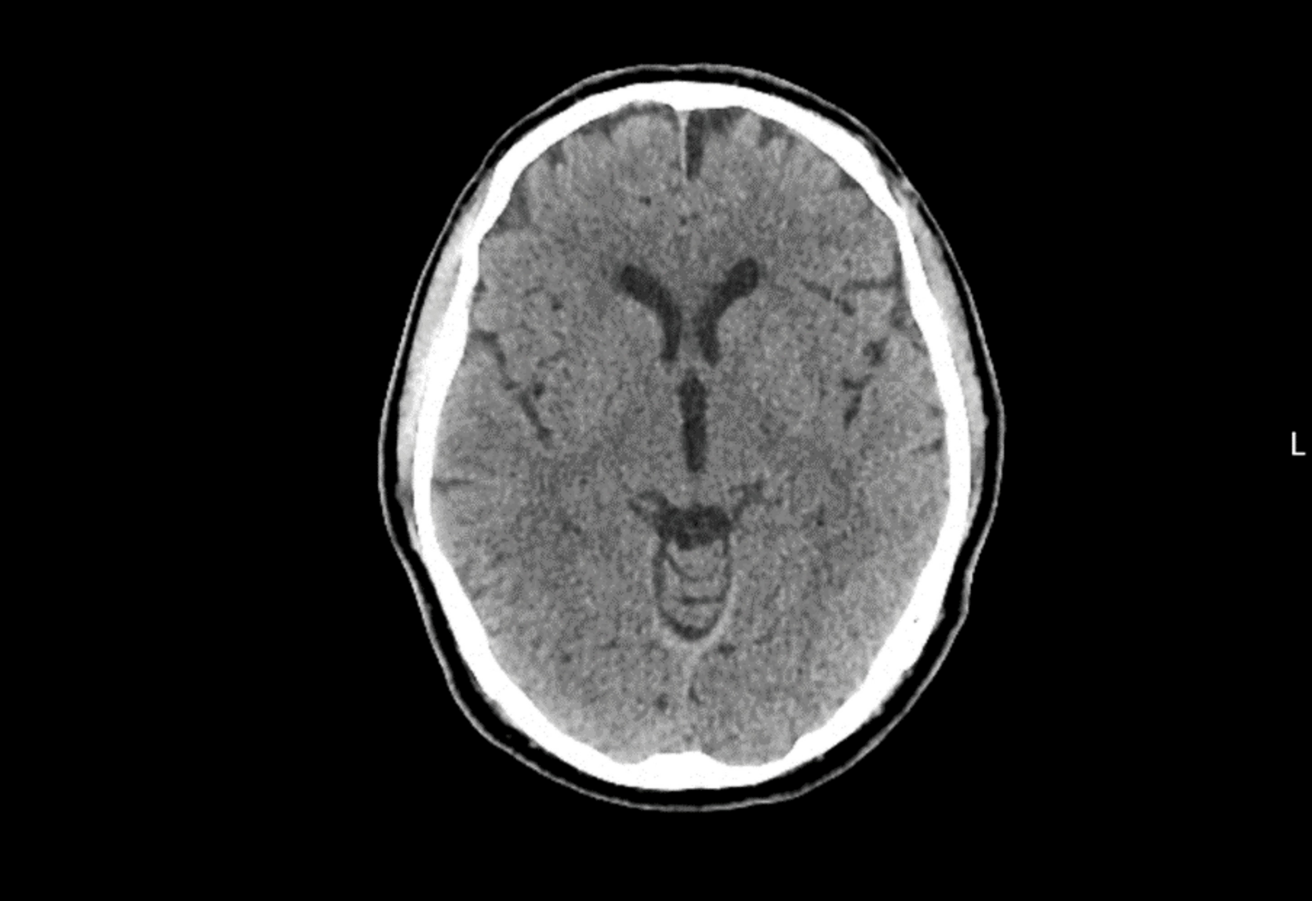
CT scan of the brain showing normal findings

Initial investigations, including a brain CT scan and blood tests, yielded no significant findings aside from the documented hypoglycemia at the time of the incident (Figure 1, Table 1). Adrenal insufficiency was ruled out based on a normal response to the short Synacthen test (Table 2). Following the event, the patient notified the DVLA and refrained from driving pending further evaluation.

**Table 1:** Laboratory data of the patient Bold values indicate significant findings.

Test	Results	Reference range
C-reactive protein	<1 mg/L	0-5 mg/L
Hemoglobin	129 g/L	130-180 g/L
White blood cell count	7 × 10⁹/L	4-11 × 10⁹/L
Platelet count	202 × 10⁹/L	150-500 × 10⁹/L
Creatinine	71 µmol/L	59-104 µmol/L
Urea	7.3 mmol/L	2.5-7.8 mmol/L
Sodium	139 mmol/L	133-146 mmol/L
Potassium	3.6 mmol/L	3.5-5.3 mmol/L
Blood glucose at the time of the incident	2.8 mmol/L	3.9-7.8 mmol/L

**Table 2:** Short Synacthen test showing a normal adrenal response

Interval	Cortisol level
0 minutes	299 nmol/l
30 minutes	568 nmol/l

Table 3 presents the results of the blood tests conducted while the patient’s blood glucose levels were being monitored.

**Table 3:** Fasting hypoglycemia with inappropriately elevated insulin and C-peptide levels Bold values indicate significant findings.

Test	Results	Reference range
Fasting blood sugar	2.7 mmol/L	The normal reference range for fasting blood sugar is 4.0-5.4 mmol/L.
C-peptide	712 pmol/L	During a hypoglycemic episode, C-peptide >300 pmol/L is inappropriately high.
Insulin	4.9 mU/L	During a hypoglycemic episode, insulin >5 mU/L is inappropriately high.

The patient was referred to the endocrinology team to rule out insulinoma. He was admitted for a supervised 72-hour fast, and the results are presented in Table 4.

**Table 4:** 72-hour supervised fasting test revealing asymptomatic hypoglycemia, accompanied by inappropriately elevated insulin and C-peptide levels Bold values indicate significant findings. HbAIc, hemoglobin A1C

Test	Results	Reference range
Blood sugar during 72 hours of supervised fasting at 14:44; asymptomatic	2.2 mmol/L	Reference range: 3.9-5.6 mmol/L
C-peptide level during low blood sugar reading in 72 hours of supervised fasting	1,350 pmol/L	During a hypoglycemic episode, C-peptide >300 pmol/L is inappropriately high.
Insulin level during low blood sugar in 72 hours of supervised fasting	14 mU/L	During a hypoglycemic episode, insulin >5 mU/L is inappropriately high.
HbA1c	32 mmol/mol	Normal: 0-41 mmol/mol

**Figure 2:**
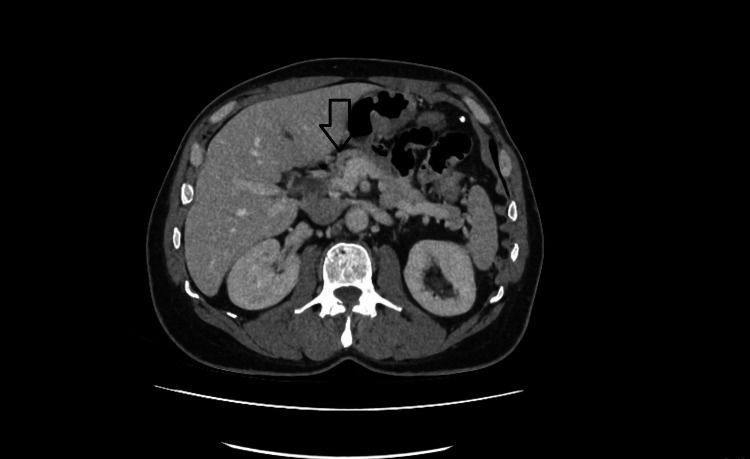
CT scan of the pancreas showing a 1.25 cm enhancing lesion in the proximal body of the pancreas

Additional imaging was performed to investigate the presence of a pancreatic lesion. A contrast-enhanced CT scan of the pancreas revealed a 1.25 cm hyperenhancing lesion in the proximal body, which was highly suggestive of an insulinoma (Figure 2).

**Figure 3:**
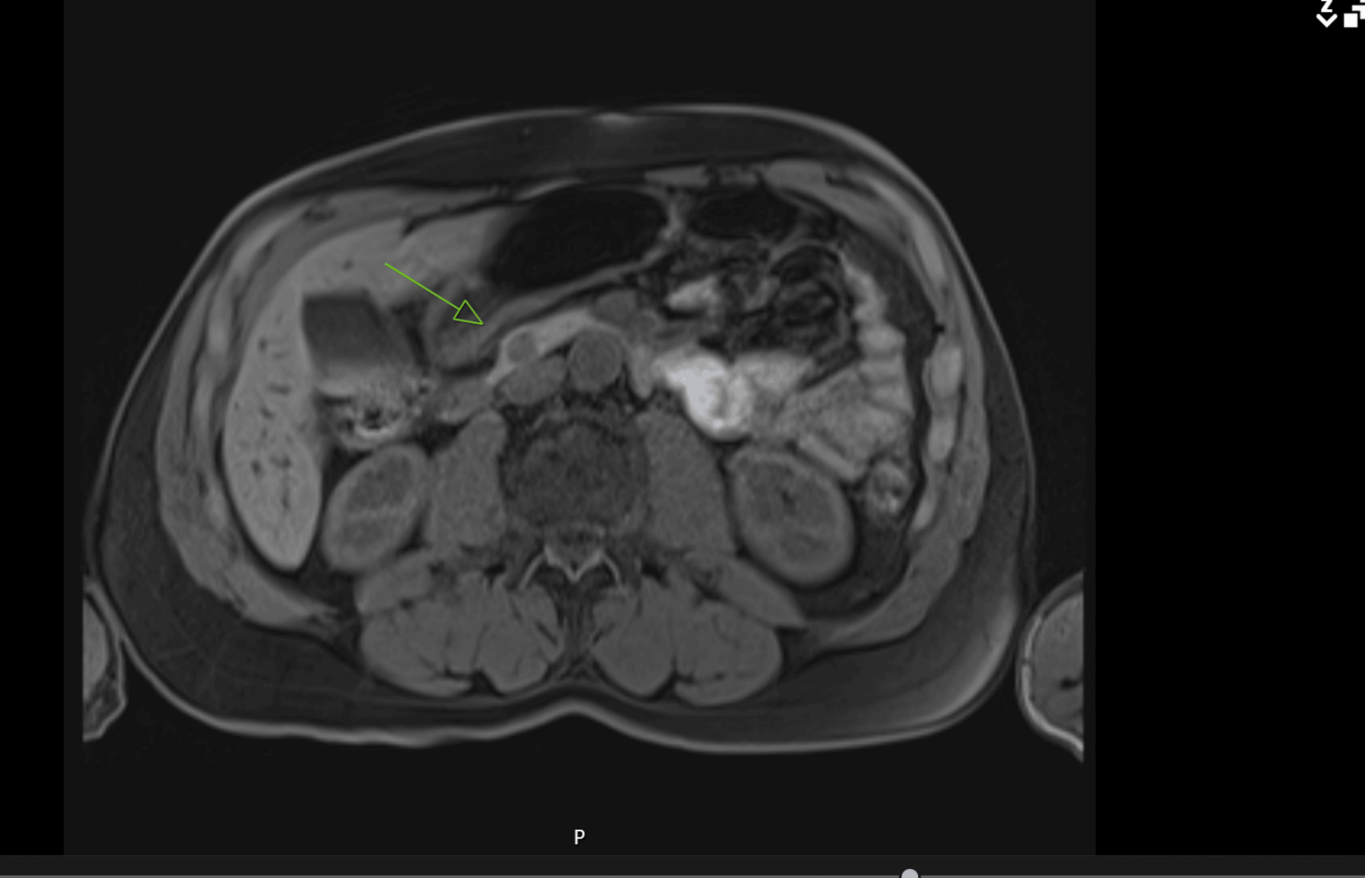
MRI of the pancreas showing a 10 mm nodule located within the inferior pancreatic head, just below the level of the ductal insertion at the ampulla

He was referred for hepatobiliary surgery, and preoperative imaging included an MRI of the pancreas, which revealed a 10 mm nodule located in the inferior pancreatic head, just below the level of the ductal insertion at the ampulla (Figure 3).

**Figure 4:**
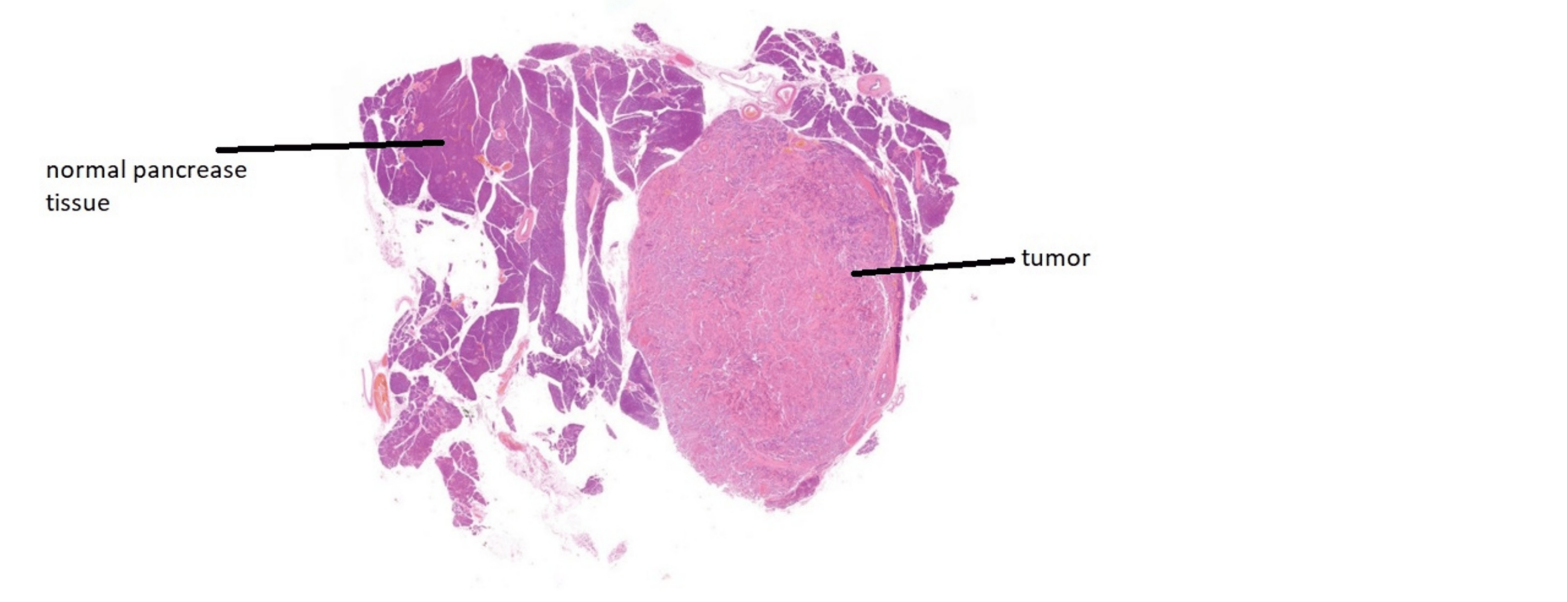
Histopathology slide showing normal pancreatic parenchyma adjacent to tumor tissue

**Figure 5:**
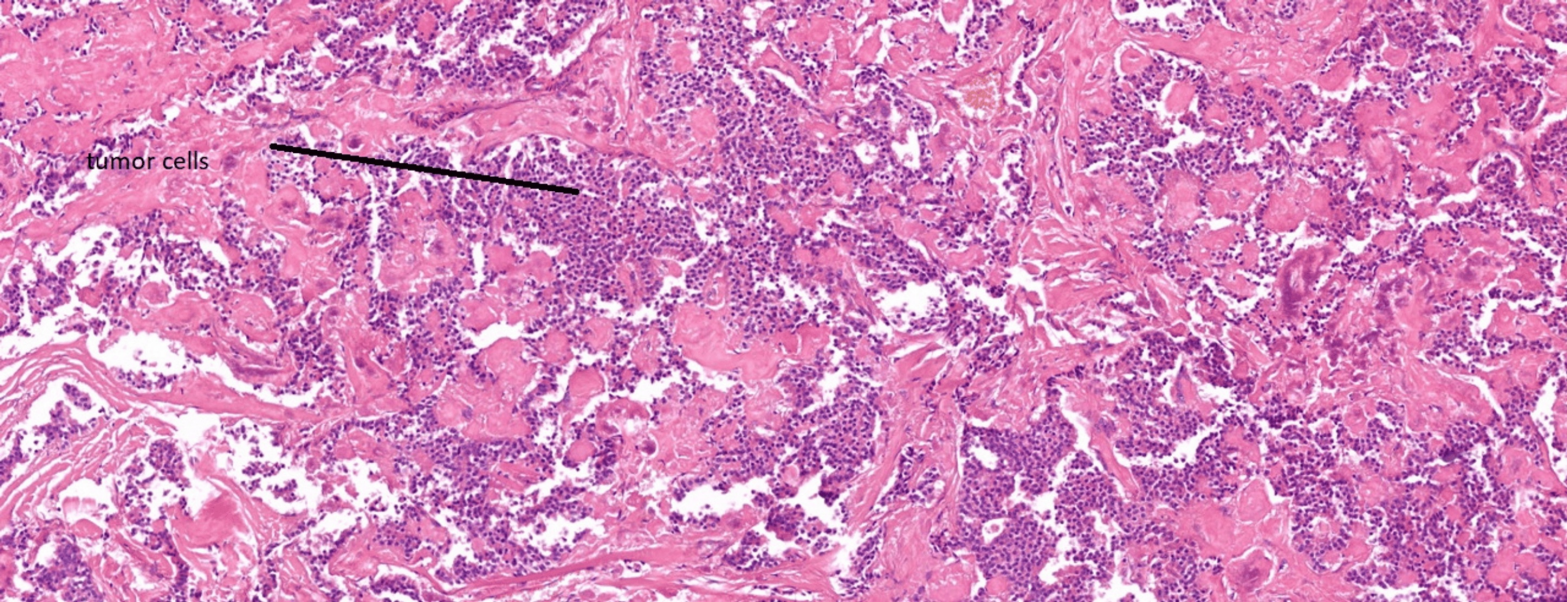
Tumor cells identified on histopathological review

The patient underwent a pylorus-preserving pancreaticoduodenectomy and cholecystectomy, collectively known as the Whipple procedure. The surgery was uncomplicated, and blood glucose levels were closely monitored throughout the perioperative period. Postoperatively, the patient was reviewed by a dietitian, and pancreatic enzyme replacement therapy was initiated to address exocrine insufficiency. Histopathological examination confirmed a grade 1, well-differentiated neuroendocrine tumor with a Ki-67 proliferation index of less than 2%, staged as pT1N0R0. The patient recovered well postoperatively and was discharged with ongoing pancreatic enzyme supplementation and regular glucose monitoring (Figure 4, Figure 5).

One month after surgery, the patient was reviewed during a follow-up appointment. He was recovering well and continued to take pancreatic enzyme supplements to support digestion, along with 40 mg of omeprazole once daily. His blood glucose levels remained stable, consistently ranging between 6 and 9 mmol/L. Several months later, he returned for a follow-up with the endocrinology team, reporting that he felt generally well. His blood sugar readings remained within normal limits, reflecting a positive outcome since his previous visit (Table 5).

**Table 5:** Laboratory tests showing a mildly elevated HbA1c within the prediabetic range, along with a normal lipid profile and vitamin D levels HbAIc, hemoglobin A1C; HDL, high-density lipoprotein; TSH, thyroid-stimulating hormone

Test	Results	Reference range
HbA1c	43 mmol/mol	0-41 mmol/mol
Cholesterol	4.7 mmol/L	0-5.2 mmol/L
Triglyceride	1.24 mmol/L	0-1.7 mmol/L
HDL	1.61 mmol/L	1.45-3 mmol/L
TSH	2.49 mU/L	0.27-4.2 mU/L
25 OH vitamin D	54 nmol/L	50-374 nmol/L

The patient submitted a follow-up application to have his driving license reinstated, which was approved, and his license was reinstated.

## Discussion

Insulinomas are rare tumors that develop in the pancreas. These tumors secrete excessive amounts of insulin, leading to a dangerous drop in blood sugar levels, which can result in episodes of hypoglycemia. Typically, individuals with hypoglycemia experience classic symptoms such as confusion, weakness, and sweating. However, one of the challenges associated with insulinomas is that some patients experience hypoglycemia unawareness. This condition prevents individuals from recognizing the usual bodily cues indicating low blood sugar, which increases their risk of severe hypoglycemic episodes [1,3].

Hypoglycemia unawareness occurs when individuals no longer perceive the typical warning signs of low blood sugar. Symptoms like shaking, sweating, and dizziness may be absent, even as blood glucose levels decrease to dangerous levels. This insensitivity typically develops over time due to repeated hypoglycemic episodes. As these events accumulate, they can dull the body’s natural counterregulatory responses, such as the release of adrenaline, which normally serves to alert individuals to an impending hypoglycemic crisis [1,3].

A study conducted at a single center involving 22 patients diagnosed with insulinomas found that nearly a third (seven individuals) exhibited hypoglycemia unawareness, suggesting that a significant proportion of insulinoma patients may present without the typical hypoglycemic symptoms [3].

Diagnosing insulinomas can be challenging due to several factors. One major issue is the misinterpretation of symptoms. When classic signs of hypoglycemia, like trembling and sweating, are absent, patients may instead present with nonspecific symptoms such as confusion, dizziness, or fainting. These can be mistaken for neurological or cardiovascular issues, leading to delays in proper diagnosis. Additionally, the presence of hypoglycemia unawareness can further complicate the recognition of insulinomas, as these fluctuating symptoms make it harder for healthcare providers to pinpoint the underlying cause [4,5].

Understanding the pathophysiology of hypoglycemia unawareness helps explain why these episodes occur. Several mechanisms are involved, primarily disruptions in glucose sensing in the brain, adaptations within the brain, and compromised hormonal counter-regulation. Normally, when blood sugar levels drop, hormones such as glucagon and epinephrine are released to help raise glucose levels. However, with repeated hypoglycemic episodes, the body’s response becomes impaired, leading to a cycle of unrecognized hypoglycemia. This shift increases the threshold for hormonal counter-regulation, contributing to hypoglycemia-associated autonomic failure, which characterizes hypoglycemia unawareness [6].

A significant study by Mitrakou et al. in 1993 further clarified these mechanisms. They demonstrated that patients with insulinomas typically have lower levels of counter-regulatory hormones, including plasma catecholamines, glucagon, growth hormone, and cortisol. In their study, which involved stepped hypoglycemic-clamp studies on six insulinoma patients compared to 14 age-, weight-, and sex-matched normal subjects, the researchers found substantial differences in hormone levels. Notably, the counter-regulatory hormone levels in insulinoma patients returned to normal following curative surgeries, highlighting the impact of insulinomas on hormonal regulation and suggesting that surgical intervention can restore normal physiological responses to hypoglycemia [7].

## Conclusions

The connection between insulinomas and hypoglycemia unawareness presents significant diagnostic challenges, as patients may exhibit nonspecific symptoms such as confusion or fainting without the typical signs of hypoglycemia. This can result in delays in both diagnosis and treatment, ultimately increasing the risk of severe complications. Prolonged and recurrent hypoglycemia can lead to serious outcomes, including seizures, coma, or even death, particularly in the absence of the usual hypoglycemic symptoms. As demonstrated in the presented case, timely recognition of insulinomas is essential to prevent the progression of hypoglycemia unawareness and associated cognitive impairments. Early diagnosis, facilitated by biochemical testing and imaging, followed by surgical resection, is crucial in restoring normal awareness of hypoglycemic events and reducing long-term risks.
